# Functional and Radiological Outcomes of Retrograde Nailing in Distal Femur Fractures: A Retrospective Study

**DOI:** 10.7759/cureus.102832

**Published:** 2026-02-02

**Authors:** Anjani Reddy Arva, Nagakumar J S, Sagar Venkataraman, Kanchuboina Gnana Kiran Theja

**Affiliations:** 1 Department of Orthopaedics, Sri Devaraj Urs Medical College, Sri Devaraj Urs Academy of Higher Education and Research, Kolar, IND

**Keywords:** distal femur fractures, functional outcomes, neer score, radiological outcomes, retrograde intramedullary nailing

## Abstract

Background: Distal femur fractures are relatively rare and complex injuries that require effective treatment. Retrograde intramedullary nailing (RIMN) has emerged as a viable alternative fixation method.

Objectives: To assess the functional and radiological outcomes of distal femur fractures treated with RIMN.

Materials and methods: A retrospective study was conducted on 40 patients who underwent RIMN for distal femur fractures in the Department of Orthopedics, RL Jalappa Hospital, Kolar, India, during the study period after retrieving data from the medical records of patients with a minimum six-month follow-up. Statistical analysis was performed using IBM SPSS Statistics software version 22 (IBM Corp., Armonk, NY). The results obtained were presented in the form of a frequency table, and continuous data were expressed as mean and standard deviation or median.

Results: The majority of participants (40%) were in the 30-40 years age group, with a mean age of 41.6 years. Males accounted for 80% of the study population. The most common fracture type was A2 (30%). Road traffic accidents (RTAs) were the leading cause of fractures (80%). The mean time for fracture union was 15.75 weeks, and the mean follow-up period was 17.65 months. Complications included nail breakage (5%), anterior knee pain (20%), and shortening of the affected limb by 1.5 cm (15%). The majority of patients (57.5%) achieved an "Excellent" outcome according to the Neer score.

Conclusion: This study demonstrates that RIMN is a highly effective treatment option for distal femur fractures. The results show a high union rate, and functional outcomes were satisfactory and good among the majority of patients according to the Neer score. The complication rate was relatively low, with nail breakage, anterior knee pain, and limb shortening being the most common issues encountered. Overall, these findings suggest that RIMN offers favorable outcomes and minimal complications, making it a reliable treatment option for distal femur fractures.

## Introduction

Distal femur fractures are uncommon, accounting for under 1% of all fractures and roughly 4%-7% of femoral fractures overall [1]. These fractures usually occur due to two different types of injury mechanisms. Older adults, particularly those with osteoporotic bone and fragile soft tissues, often experience these fractures due to low-energy trauma, such as falls or sprains, with a high rate of comorbidities. This demographic is predominantly female, with 60% of patients being women over the age of 60. In contrast, young patients, typically male (60%) and under the age of 40, tend to sustain distal femur fractures due to high-energy trauma, resulting in complex injuries with comminuted and open fracture patterns. Notably, approximately 30% of patients with distal femur fractures present with polytrauma [2].

Managing distal femur fractures presents difficulties because of their nearness to neurovascular elements, which heightens the likelihood of vascular damage. Additionally, these fractures are situated near the articular surface of the knee joint, which can result in early impairment of joint mobility and necessitate precise management to prevent long-term functional limitations [3]. Traditionally, antegrade nailing has been considered the gold standard for treating certain fractures. However, retrograde nailing has emerged as a viable alternative fixation method. Over the past two decades, retrograde nailing has gained popularity, particularly in North America. This minimally invasive procedure involves inserting the nail through the distal femur's intercondylar notch, thereby preserving blood supply and fracture hematoma while minimizing significant soft tissue damage [4].

The outcome after management is affected by bone quality, patient's age, articular surface involvement, size of fragments, fixation device, and soft tissue injuries [5]. The overall incidence of distal femur fractures was 8.7/100,000/year. After the age of 60 years, a rapid increase in the incidence of distal femoral fractures was observed in both genders, with a large female predominance. Low-energy injuries were the most common mode of injury in both genders (97%), with approximately 61% being the result of a fall from standing height [6].

Literature has not adequately explored the benefits of retrograde intramedullary nailing (RIMN) in achieving optimal outcomes for distal femur fractures, specifically regarding fracture healing, callus formation, angular alignment, and early postoperative recovery. Hence, the present study aimed to assess the functional and radiological outcomes of distal femur fractures treated with RIMN. Specifically, the study focused on two key areas: radiological outcomes, as evidenced by fracture union on follow-up X-rays, and functional outcomes, as measured by the Neer score [7].

## Materials and methods

This retrospective observational study was conducted on 40 patients who underwent RIMN for distal femur fractures in the Department of Orthopaedics at R.L. Jalappa Hospital and Research Centre, Kolar, India, over a five-and-a-half-year period, from January 2018 to June 2023. The study aimed to analyze clinical, radiological, and functional outcomes in patients who underwent retrograde intramedullary nailing for distal femur fractures.

Study design and sampling technique

A retrospective census sampling technique was employed. All eligible patients who underwent the procedure during the study period and met the inclusion criteria were included. No randomization or sampling was done, as all qualifying records were reviewed to avoid selection bias.

Inclusion criteria

The inclusion criteria were as follows: Patients aged 18 years and above; patients diagnosed with distal femoral fractures classified as AO Foundation/Orthopaedic Trauma Association type 33A; patients who underwent RIMN between January 2018 and June 2023; patients with a minimum follow-up period of six months; patients with complete and accessible medical records.

Exclusion criteria

The exclusion criteria were patients with multiple fractures or open fractures; pathological fractures or old fractures (defined as time from injury to surgery exceeding 21 days); non-ambulatory patients or those unable to undergo surgery due to frailty or severe comorbidities; and cases with incomplete data or inadequate follow-up documentation.

Data collection

Data were retrieved retrospectively from patient medical records and surgical logs. The following parameters were documented: Demographics: age, gender; Clinical details: fracture classification, mechanism of injury, articular and soft tissue involvement; Operative details: duration of surgery, intraoperative observations; Postoperative outcomes: surgical site pain, complications (e.g., infection, implant failure); Radiological outcomes: fracture union assessed via follow-up X-rays; Functional outcomes: assessed using the Neer scoring system, a validated tool for evaluating knee function post-intervention [7].

Statistical analysis

Data was analyzed using IBM SPSS Statistics software, version 22 (IBM Corp., Armonk, NY). Categorical variables were presented as frequencies and percentages. Continuous variables were expressed as means and standard deviations (for normally distributed data) or medians and interquartile ranges (for skewed data). Appropriate statistical tests, such as the chi-square test for categorical variables and Student’s t-test or Mann-Whitney U test for continuous variables, were employed depending on the distribution and nature of the data. A p-value < 0.05 was considered statistically significant.

## Results

The distribution of study participants based on age is presented in Table 1. Subjects were divided into four age groups: less than 30 years, 30-40 years, 41-50 years, and over 50 years. The majority of participants (16, 40%) were in the 30-40 years age group, followed by 11.11 (27.78%) in the 41-50 years group and 8.88 (22.22%) in the over 50 years category. The smallest group consisted of individuals under 30 years, comprising four (10%) of the total. In total, there were 40 participants, with a mean age of 41.6 years and a standard deviation of 9.62 years. 

**Table 1:** Age-wise distribution of the study subjects The majority of subjects were in the 30–40 years age group (16, 40%), followed by 41–50 years (11.11, 27.78%). The mean age of the study population was 41.6 ± 9.62 years.

Age group (years)	Frequency (n)	Percentage (%)
<30	4	10.00
30-40	16	40.00
41-50	11	27.78
>50	9	22.22
Total	40	100.00
Mean age (years)	41.6+9.62	

Table 2 categorizes participants based on gender. Males were the predominant group, accounting for 32 (80%), while females represented eight (20%). These data highlight a significant gender imbalance among the study subjects.

**Table 2:** Gender-wise distribution of the study subjects The majority were males (32, 80%), while females constituted eight (20%) of the study population.

Gender	Frequency (n)	Percentage (%)
Male	32	80.00
Female	8	20.00

Participants were grouped based on the type of fracture (Table 3). The most common types were A2 fractures, found in 12 (30%) of cases, followed by A1 fractures (11, 27.5%) and A3 fractures (7, 17.5%). C1 fractures accounted for eight (20%), while the least common were C2 fractures at two (5%). Additionally, fractures were classified as either simple or compound, with simple fractures being more prevalent (25, 62.5%) compared to compound fractures (15, 37.5%). 

**Table 3:** Type of fracture-wise distribution of study subjects This table demonstrates the distribution of fractures among study participants based on AO Foundation classification and fracture type. The most common fracture pattern was A2 (12, 30%), followed by A1 (11, 27.5%). Overall, simple fractures (25, 62.5%) were more frequent than compound fractures (15, 37.5%).

Type of fracture	Frequency	Percentage
A1	11	27.5
A2	12	30.00
A3	7	17.5
C1	8	20.00
C2	2	5.00
Fracture wise		
Simple	25	62.5
Compound	15	37.5

Table 4 identifies the modes of injury leading to fractures among the participants. Road traffic accidents (RTAs) were the leading cause, responsible for 32 (80%) of the cases. Falls accounted for the remaining eight (20%). These data underscore the significant role of RTAs in causing fractures.

**Table 4:** Mode of Injury wise distribution of study subjects This table shows the mechanism of injury among study participants. Road traffic accidents (RTA) were the predominant cause 32(80%), whereas falls accounted for 8(20%)of cases.

Mode of Injury	Frequency	Percentage
Fall	8	20.00
RTA	32	80.00

Table 5 highlights the side of the body affected by fractures. Right-sided fractures were more common, comprising 26 (65%) of the cases, while left-sided fractures occurred in 14 (35%) of participants.

**Table 5:** Distribution of the study subjects based on the side of fracture The right side was more commonly involved (26, 65%) compared to the left side (14, 35%).

Mode of Injury	Frequency (n)	Percentage (%)
Right	26	65.00
Left	14	35.00

The operative parameters are summarized in Table 6 as mean values with standard deviations. Blood loss during surgery averaged 482.5 ml with a standard deviation of 222.90 ml. The average duration of surgery was 66.25 minutes (SD = 15.22 minutes). The mean time for fracture union was 15.75 weeks (SD = 2.03 weeks), while the mean follow-up period was 17.65 months (SD = 5.11 months). The knee range of motion (ROM) among participants varied from 80 to 130 degrees.

**Table 6:** Operative parameters among subjects This table summarizes the intraoperative and postoperative parameters of the study population. The mean blood loss was 482.5 ± 222.9 ml, with an average operative time of 66.3 ± 15.2 minutes. The mean time to union was 15.8 ± 2.0 weeks, and the mean follow-up duration was 17.7 ± 5.1 months. The postoperative knee range of motion (ROM) ranged from 80° to 130°.

Parameters	Mean+SD
Blood loss (ml)	482.5+222.90
Duration of surgery (min)	66.25+15.22
Time of union (weeks)	15.75+2.03
Follow-up (months)	17.65+5.11
Knee ROM (range)	80-130 degrees

Complications observed in the study subjects (Table 7) included nail breakage (5%), anterior knee pain (20%), and shortening of the affected limb by 1.5 cm (15%). These findings illustrate the postoperative challenges faced by the participants. 

**Table 7:** Postoperative complications observed in the study population The most frequent complication was anterior knee pain (8, 20%), followed by limb shortening of 1.5 cm (6, 15%) and nail breakage (2, 5%).

Complications	Frequency (n)	Percentage (%)
Nail breakage	2	5.00
Anterior knee pain	8	20.00
1.5 cm shortening	6	15.00

The Neer score distribution among participants reflects their recovery outcomes (Table 8) [7]. The majority (23, 57.5%) achieved an "Excellent" outcome, while 11 (27.5%) were classified as "Good." A smaller proportion had "Fair" (four, 10%) and "Poor" (two, 5%) outcomes, indicating varied recovery success across the cohort. 

**Table 8:** Neer score of the patients This table presents the functional outcomes of the study population assessed using the Neer scoring system [7]. The majority of patients achieved excellent results (23, 57.5%), followed by good (11, 27.5%), fair (four, 10%), and poor outcomes (two, 5%).

Neer score	Frequency (n)	Percentage (%)
Poor	2	5.00
Good	11	27.5
Fair	4	10.00
Excellent	23	57.5

## Discussion

Different plating alternatives, such as fixed- and variable-angle blade plates, have been suggested for addressing distal femur fractures. However, these plates are linked to increased occurrences of malunion, nonunion, implant failure, and infection because of their rigid structure and the consequent soft tissue damage. The emergence of minimally invasive plate osteosynthesis techniques has lessened these issues to a certain degree. These plates utilize the internal fixator principle, allowing fracture union through callus formation due to the relative stabilization achieved. Alternatively, retrograde intramedullary nailing offers the advantage of being a load-sharing device that bypasses the fracture site, providing a minimally invasive approach [8]. Hence, the present study aimed to evaluate the functional and radiological outcomes of distal femur fractures treated with RIMN.

The demographic characteristics of the study participants revealed a mean age of 41.6 years, with a standard deviation of 9.62. This suggests that the majority of participants were middle-aged adults. Additionally, the study group was mainly composed of males, accounting for 80% (32 individuals) of the overall sample. According to Shah et al., the average age of patients was 58.41 ± 4.21, which was greater than that of our study cohort [8]. Another study by Shah et al. reported a mean age of 35.20 ± 10.66 years, which is a lower age group than our study; however, the gender difference corresponded to our study, with predominantly male patients, accounting for 74 (76.3%) of the total, while female patients comprised 23 (23.7%) of the sample [9]. Raza A et al. also reported similar results. This gender disparity may be attributed to various factors, including occupational hazards, lifestyle, or other environmental influences.

The fracture patterns observed in this study revealed that A2 fractures were the most common, accounting for 30% of cases, followed by A1 fractures (11, 27.5%) and A3 fractures (seven, 17.5%). C1 fractures comprised eight (20%) of cases, while C2 fractures were the least common, occurring in only two (5%) of patients. The observed distribution of fracture patterns aligns with prior research, emphasizing the significance of accurate classification in influencing treatment choices. Shah et al. have indicated that within the AO-OTA classification system, the most frequently observed type of distal femur fracture is 33A2, representing 36 (50.7%) of the total, which is consistent with our findings, followed by 33A3 at 23 (32.3%), with 33A1 being the least prevalent at 12 (17%) [8].

The modes of injury leading to fractures were predominantly RTA, which accounted for 80% of cases. Falls were responsible for the remaining 20%. This finding underscores the significant role of RTAs in causing fractures and highlights the need for continued efforts to improve road safety. Similar to our study, Shah et al. also found RTAs as the most common mechanism of injury, accounting for 42 (59.15%) of the total. Shah et al. also reported similar findings [9].

An analysis of the side of the body affected by fractures revealed that right-sided fractures were more common, occurring in 26 (65%) of cases, while left-sided fractures occurred in 35% of patients. Our results are in accordance with Shah et al. [9], who observed that 60 (61.9%) had fractures on the right side, while 37 (38.1%) had fractures on the left side [9]. Comparable results have been noted by Raza et al. [4]. This observation could be linked to several factors, such as the usage of the dominant hand or leg, and requires additional research to assess its importance.

RIMN is recommended for managing distal femur fractures that fulfill certain criteria. These criteria include type A fractures according to the Müller classification, open wounds near the knee, and injuries that necessitate a supine position with the thorax and head elevated. Furthermore, RIMN is appropriate for patients with bilateral femur fractures, ipsilateral multilevel fractures, which may include proximal femur or combined femur and tibia fractures, periprosthetic fractures around total knee arthroplasty, and cases involving severe obesity that may complicate other treatment methods [3].

The operative parameters of the present study reported that the average blood loss during surgery was 482.5 ml, and the average duration of surgery was 66.25 minutes. These findings suggest that the surgical procedure is relatively straightforward, with manageable blood loss and operative time. The mean time for fracture union was 15.75 weeks, and the mean follow-up period was 17.65 months, indicating a relatively rapid recovery process. In contrast to our study, Shah et al. observed a notably longer average surgical duration, which was recorded at 112.34 ± 13.8 minutes [7]. In a study conducted by Prabhakaran et al. [3] involving 28 patients who underwent RIMN, the mean time to achieve union was 10.5 weeks. Surgical metrics included an average operative time of 89.64 minutes (with a range of 70-180 minutes) and an average blood loss of 224.29 ml.

The increased likelihood of union seen in patients undergoing intramedullary nailing can be linked to the release of medullary contents at the fracture location during the insertion of the nail. This process may stimulate an enhanced healing response. Additionally, the intramedullary stabilization provided by the nail, combined with a long working length, may also contribute to accelerated union [10]. This notion is supported by a study conducted by Henderson et al. [11], which demonstrated a significantly greater amount of callus formation at the fracture site 12 weeks post fixation in patients treated with intramedullary nailing compared to those treated with plating, with a two- to four-fold increase in callus formation observed in the nailed patients.

Complications observed in the study subjects included nail breakage (two, 5%), anterior knee pain (eight, 20%), and shortening of the affected limb by 1.5 cm (six, 15%). These findings highlight the potential challenges and complications associated with the surgical management of distal femur fractures. However, the relatively low incidence of these complications suggests that the procedure is generally safe and effective. In concurrence with our study, Hierholzer et al. [2] reported low complication rates in distal femur fractures treated with RIMN, including hematoma formation (one patient), superficial infection (one patient), and secondary bone grafting required in three (7%) of cases. The study by Shah et al. reported postoperative superficial infections in 8.1% (3 cases) and knee stiffness in two (5.4%) [8]. In a study assessing knee function after surgery, Prabhakaran et al. [3] reported a ROM between 0 and 81 degrees, with an average American Knee Society Score of 70.36%. Complications were observed in 16 (42.8%) of the patients, which included knee pain (5, 14.3%), knee stiffness (two, 7.1%), shortening (five, 14.3%), and infection (two, 7.1%). Notably, 22 (57.2%) of patients experienced no complications. Leg length discrepancy was observed, with eight cases exhibiting shortening of less than 1 cm and 4 cases with shortening of 2 cm or more. A systematic review by Aggarwal et al. [12] also supported the present study’s findings, reporting that both RIMN and locking plates (LP) exhibit comparable outcomes in terms of fracture union time, overall complication rates, reoperation rates, and surgical duration. These findings suggest that while RIMN may offer advantages in reducing nonunion and infection rates, LP provides better knee mobility, and the choice between the two options should be tailored to individual patient needs.

In the present study, the Neer score [7] distribution among participants reflects their recovery outcomes, with 23 (57.5%) achieving an "Excellent" outcome, 11 (27.5%) classified as "Good," four (10%) as "Fair," and two (5%) as "Poor." These findings indicate that the majority of patients achieved satisfactory recovery outcomes, with a significant proportion experiencing excellent results. This suggests that the surgical management of distal femur fractures can be highly effective in restoring function and promoting recovery. A study conducted by Hierholzer et al. [2] demonstrated that RIMN is a highly effective treatment option for distal femur fractures. Clinical and radiographic evaluations revealed that over 90% of patients achieved osseous healing within six months following RIMN. Moreover, a comparative analysis with locking compression plate (LCP) plating showed no statistically significant differences in terms of osseous healing, nonunion rates, and postoperative complications, suggesting that RIMN is a reliable and efficient treatment method for distal femur fractures, offering comparable outcomes to LCP plating. Shah et al. evaluated patients according to the Schatzker and Lambert criteria and reported that 48.6% of patients achieved excellent results and 11 (29.8%) had good results [8].

Additionally, in line with our research, Handolin et al. [13] carried out a retrospective analysis involving 44 consecutive patients with 46 distal femoral fractures treated using a RIMN (distal femoral nail (DFN)). The findings indicated a high union rate of 38 (95%), with an average union time of 17.5 weeks (ranging from eight to 68 weeks), suggesting that the DFN serves as a dependable option for managing distal femoral fractures, accompanied by a low rate of complications. However, in their study, two cases exhibited inadequate restoration of limb axial alignment and length. In three instances, there was a loss of reduction, and one case resulted in non-union. Furthermore, two cases of breakage in the distal locking screw were noted. One patient experienced an iatrogenic injury to a branch of the deep femoral artery. Although there were no occurrences of deep infections, three superficial infections were documented. Similarly, a study by Gao et al. compared RIMN with locked plating for the treatment of distal femur fractures, and no differences were found with respect to postoperative malreduction, deep infection, hardware failure, operating time, knee pain, Hospital for Special Surgery Knee Score, and ROM of the knee [14]. Another study by Demirtas et al. [15] conducted a comparative analysis on the treatment of extra-articular distal femur fractures using retrograde intramedullary nailing and bridge plating. Their research indicated no notable differences between the two treatment methods concerning implant failure, malunion, non-union, knee pain, or time to union. Gill et al. evaluated the outcomes of locked compressive plating versus retrograde nailing in treating femur fractures and reported that the complication rates were comparable across both groups. They found that the mean time to union was shorter in the retrograde nail group, averaging 22.6 weeks (SD=13.1; range 12-60 weeks), in contrast to the locked plating group, which had an average of 26.5 weeks (SD=12.9; range 12-64 weeks). Furthermore, the overall union rates were similar, and the functional scores were similar between the two methods, suggesting that both approaches provide comparable clinical outcomes.

## Conclusions

To conclude, the present study demonstrated a high union rate, with a mean time to fracture union of 15.75 weeks, and satisfactory functional outcomes, with 23 (57.5%) patients achieving an "Excellent" outcome according to the Neer score. The rate of complications was quite low, with the most frequently encountered problems being nail breakage, anterior knee discomfort, and shortening of the limb. These results indicate that RIMN is a dependable and efficient treatment choice for fractures of the distal femur, providing positive results and few complications.
